# Crystal structures of *N*-[4-(tri­fluoro­meth­yl)phen­yl]benzamide and *N*-(4-meth­oxy­phen­yl)benz­amide at 173 K: a study of the energetics of conformational changes due to crystal packing

**DOI:** 10.1107/S2056989022000950

**Published:** 2022-02-08

**Authors:** Wayne H. Pearson, Joseph J. Urban, Amy H. Roy MacArthur, Shirley Lin, Dylan W. L. Cabrera

**Affiliations:** a Chemistry Department, United States Naval Academy, 572 Holloway Rd, Annapolis, MD 21402, USA

**Keywords:** crystal structure, inter­molecular forces, aryl amides, DFT calculations, Hirshfeld surfaces, mol­ecular inter­action energies

## Abstract

As a part of our study of the syntheses of aryl amides, the crystal structures of two benzamides were determined from single-crystal X-ray data at 173 K. Both crystal structures contain mol­ecular units as asymmetric units with no solvent in the unit cells. Crystal structure I, **TFMP**, is the result of the crystallization of *N*-[4-(tri­fluoro­meth­yl)phen­yl]benzamide, C_14_H_10_F_3_NO. Crystal structure II, **MOP**, is composed of *N*-(4-meth­oxy­phen­yl)benzamide, C_14_H_13_NO_2_, units. **TFMP** is triclinic, space group *P*  , consisting of two mol­ecules in the unit cell related by the center of symmetry. **MOP** is monoclinic, space group *P*2_1_/*c*, consisting of four mol­ecules in the unit cell. Both types of mol­ecules contain three planar regions; a phenyl ring, an amide planar region, and a *para*-substituted phenyl ring. The orientations of these planar regions within the asymmetric units are compared to their predicted orientations, in isolation, from DFT calculations. The aryl rings are tilted approximately 60° with respect to each other in both experimentally determined structures, as compared to 30° in the DFT results. These conformational changes result in more favorable environments for N—H⋯O hydrogen bonding and aryl ring π-stacking in the crystal structures. Inter­molecular inter­actions were examined by Hirshfeld surface analysis and qu­anti­fied by calculating mol­ecular inter­action energies. The results of this study demonstrate that both hydrogen bonding and dispersion are essential to the side-by-side stacking of mol­ecular units in these crystal structures. Weaker dispersion inter­actions along the axial directions of the mol­ecules reveal insight into the melting mechanisms of these crystals.

## Abstract 2

The conformations of two aryl amides have been determined experimentally in crystal structures using X-ray data and calculated with DFT methods for the isolated mol­ecules. Geometrical comparisons are made along with energy analyses of the inter­molecular inter­actions in the two crystal structures.

## Chemical context

Numerous methodologies have been developed to form amide C—N bonds due to their prevalence in biomolecules, such as peptides and proteins, and in synthetic targets (Seward & Jakubke, 2002 ▸; Greenberg *et al.*, 2000 ▸). In particular, aryl amides can be found in a variety of pharmaceutical drugs and in polymers such as Kevlar^TM^ (Masse *et al.*, 1998 ▸; Evano *et al.*, 2004 ▸, 2008 ▸; Satyanarayana *et al.*, 2007 ▸; Tanner *et al.*, 1989 ▸). A series of aryl amides were synthesized and isolated during the development of a copper-mediated concurrent tandem catalytic methodology for the amidation of aryl chlorides (Chang *et al.*, 2019 ▸). The crystal structures of two of these aryl amides, derived from the cross-coupling of either 4-chloro­benzotrifluoride or 4-chloro­anisole with benzamide, are reported here. 
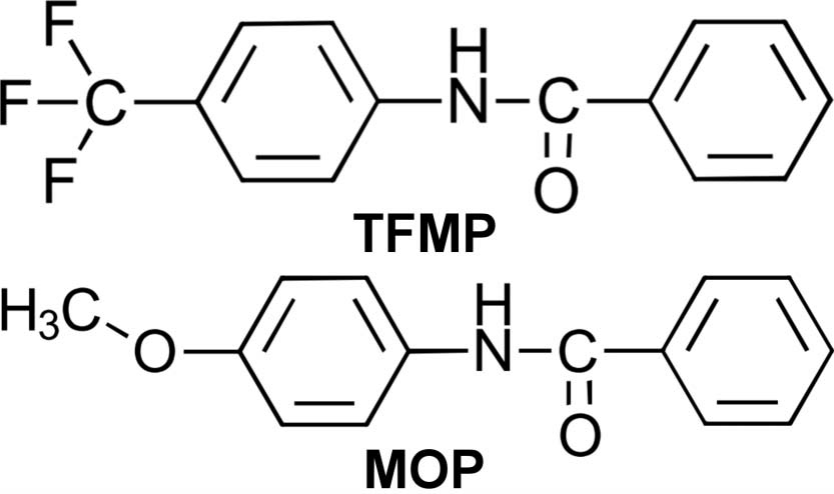


## Structural commentary

**Figure 1:**
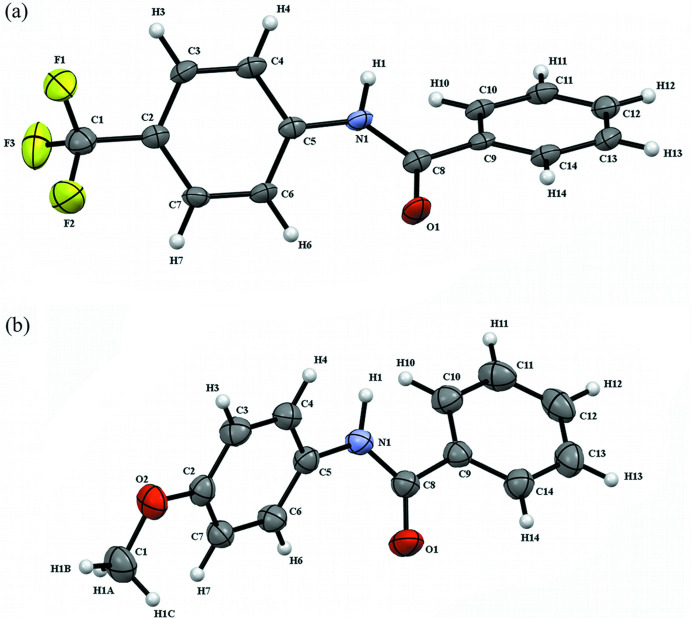
The mol­ecules present in the asymmetric units in (*a*) **TFMP** and (*b*) **MOP**. Displacement ellipsoids are drawn at the 50% probability level. Hydrogen atoms are represented by spheres of 0.20 Å radius.

The reported compounds are substituted benzamides containing a *para*-substituted phenyl ring in place of one of the hydrogen atoms of the amide nitro­gen. In both crystal structures, the asymmetric unit is a single mol­ecule of the compound. Crystal structure I, **TFMP**, contains an asymmetric unit with a tri­fluoro­methyl­phenyl ring. Crystal structure II, **MOP**, has an asymmetric unit with a meth­oxy­phenyl ring. The mol­ecular structures in the form of ellipsoid plots are shown in Fig. 1 ▸. There is nothing remarkable about the individual bond lengths, bond angles, or planarity of the aryl rings in these mol­ecules.

**Figure 2:**
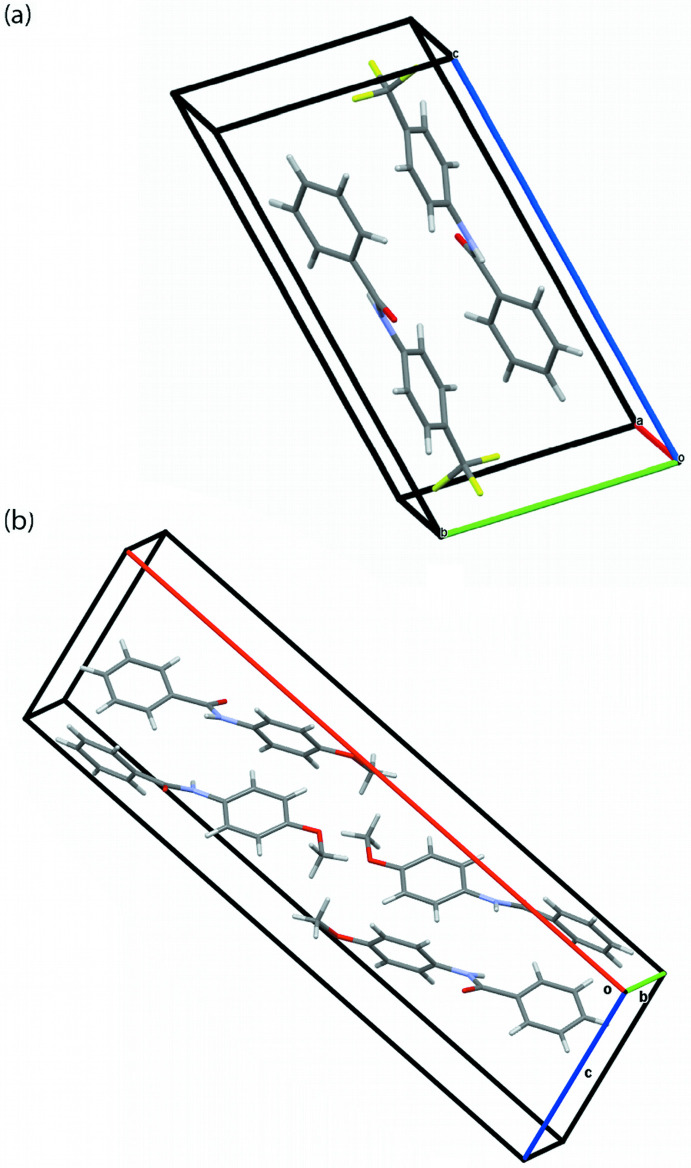
Unit-cell packing of (*a*) **TFMP** and (*b*) **MOP**.

Fig. 2 ▸ contains the unit cells for both crystal structures. Both mol­ecules assume chiral configurations. Because the space groups are centrosymmetric, the unit-cell contents are racemic mixtures containing the enanti­omers of the mol­ecules in symmetry-related positions. In both crystal structures, the mol­ecules align along the mol­ecular axes. This alignment results in the long axes in both unit cells, *c* = 14.415 (3) Å in **TFMP** and *a* = 26.783 (2) Å in **MOP**.

**Figure 3:**
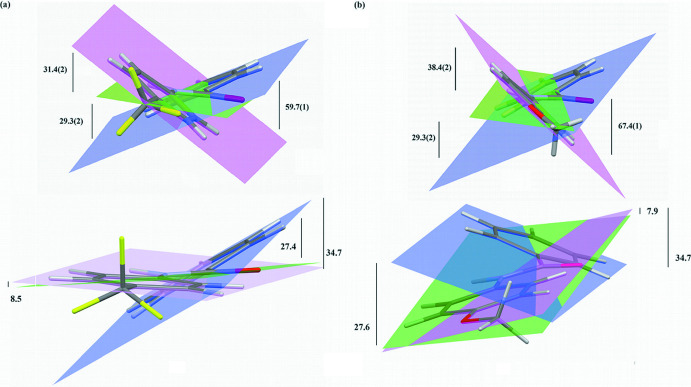
Views of orientations of planar regions and their dihedral angles (in °) from experimental results (top) and DFT calculations (bottom) for (*a*) **TFMP** and (*b*) **MOP**. Blue = phenyl ring; green = amide plane; mauve = *para*-substituted phenyl ring.

Both mol­ecules contain three planar regions; a phenyl ring, an amide linkage, and the *para*-substituted phenyl ring. Rotation of the rings relative to each other can lead to conformations that exist in the crystal structures that differ from the native mol­ecular conformations. The relationship between the conformations of organic mol­ecules and crystal structures has been reported extensively and summarized in the review article by Cruz-Cabeza & Bernstein (2014 ▸). Tilt angles were determined by comparing the angles between normals to least-squares planes as defined by the non-hydrogen atoms in a planar region. Significant tilt angles exist between the planar regions in both mol­ecules in the experimentally determined structures as shown in Fig. 3 ▸.

## DFT calculations and results for isolated mol­ecules

Quantum-chemical density functional theory (DFT) calculations were performed to find the conformations of global minimum energy for the two mol­ecules in isolation. Calculations were performed with the *GAUSSIAN09* (Frisch *et al.*, 2016 ▸) program suite on DoD High Performance Modernization resources. Initial conformer searching was performed at the mol­ecular mechanics level with the MMFF force field as implemented in *SPARTAN* mol­ecular modeling software (Wavefunction, 2014 ▸). Viable structures were then subjected to complete geometry optimizations in *GAUSSIAN09* at the M06-2X/6-31+G(d) level (Zhao & Truhlar, 2008 ▸). Frequency calculations were performed at M06-2X/6-31+G(d) to confirm that all stational points were minima. Comparisons of bond lengths and angles between the experimentally determined structures and the DFT calculations can be found in the supporting information.

Tilts of the planar regions from the DFT calculations are also shown in Fig. 3 ▸. The amide plane/phenyl ring angles are approximately 29° in the experimental results and 27° in the calculated mol­ecules. In the experimentally determined structures, the angles between *para*-substituted phenyl rings and the amide planes are 31.4 (2)° in **TFMP** and 38.4 (4)° in **MOP**. The DFT calculations yield much smaller angles of 8.5 and 7.9°, respectively. These results indicate that the conformational change due to crystal packing in both mol­ecules is primarily due to ring tilts around the N1—C5 bonds while the rings joined by C8—C9 bonds are essentially oriented the same as in the isolated mol­ecules. A search of benzamides in the Cambridge Structural Database (version 2020.3; Groom *et al.*, 2016 ▸) revealed a number of compounds with similar phenyl ring/amide plane tilts. For example, *N*-phenyl­benzamide (Wang *et al.*, 2014 ▸), *N*-(4-hy­droxy­phen­yl)benzamide (Tothadi & Desiraju, 2012 ▸), benzamide (Blake & Small, 1972 ▸) and *N*-(4-nitro­phen­yl)benzamide (du Plessis *et al.*, 1983 ▸) all possess amide plane/phenyl ring angles between 28 and 31°. A likely explanation for the consistent amide plane/phenyl ring tilt would be the balance of the attractive O1⋯H14 inter­actions and the repulsive inter­actions of H1⋯H10.

Additional DFT calculations were performed to determine approximate energy differences between the mol­ecules in isolation and conformations found in the crystal structures. To best approximate the conformations in the experimentally determined structures, dihedral angles around the amide linkage were constrained to crystallographic values while all other geometrical parameters were allowed to vary. Tilt angles between phenyl and *para*-substituted phenyl rings are in good agreement between the X-ray models and DFT calculations. For **TFMP**, the angles are 59.7 (1)° in the crystal structure and 59.6° in the DFT calculation. For **MOP**, the angles are 67.4 (1)° in the crystal structure and 66.8° in the DFT calculation. The results of the DFT calculations show that the energies of the conformations in the experimentally determined structures are slightly above those in the isolated mol­ecules, *viz*. 3.2 kJ mol^−1^ higher for **TFMP** and 2.5 kJ higher for **MOP**.

## Supra­molecular features

**Figure 4:**
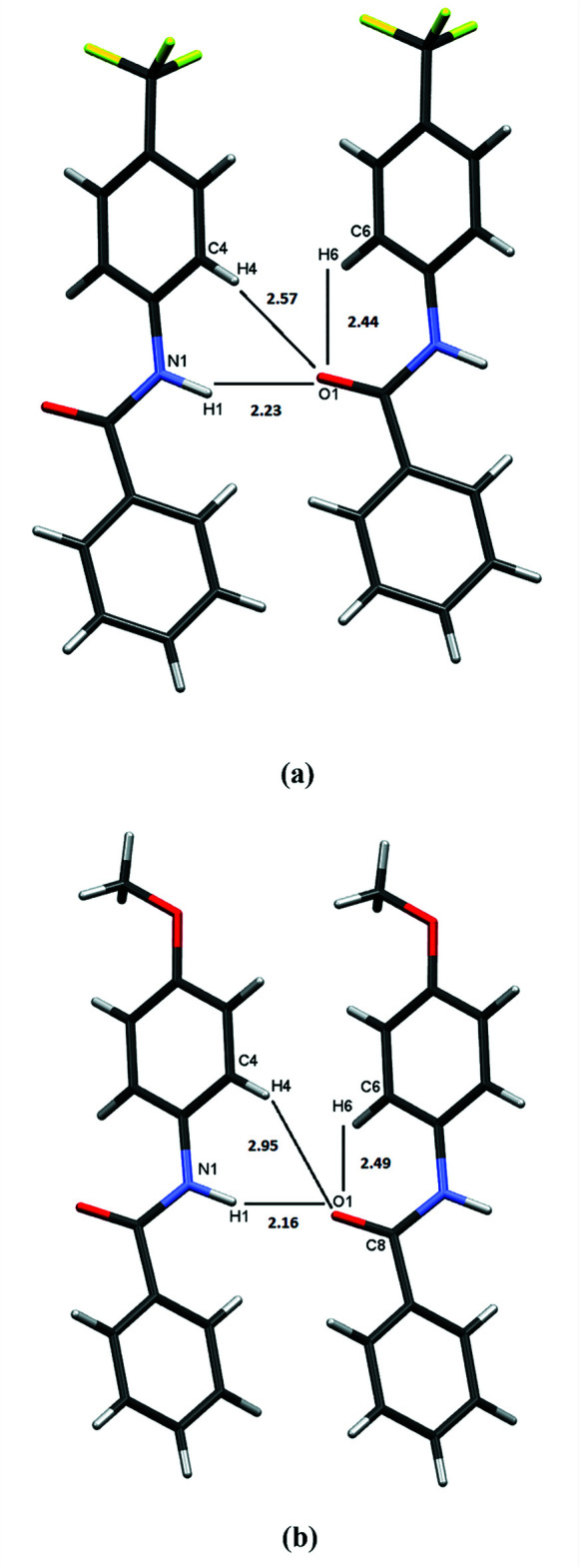
Hydrogen bonding contacts (in Å) in (*a*) **TFMP** and (*b*) **MOP**.

Close packing in both crystal structures is the result of hydrogen bonding, dipole inter­actions and dispersion. Hydrogen bonds were revealed by using the HTAB command in *SHELXL* (Sheldrick, 2015*b* ▸) and verified using *PLATON* (Spek, 2020 ▸). The H⋯O contacts are listed in Tables 1 ▸ and 2 ▸ and shown in Fig. 4 ▸. There is only one type of N—H⋯O inter­action in both crystal structures, in the direction parallel to the *a* axis for **TFMP** and the *b* axis in **MOP**. There are non-classical carbon-based hydrogen bonds that exist as intra­molecular inter­actions (C6—H6⋯O1) in both crystal structures and inter­molecular contacts (C4—H4⋯O1) in **TFMP** only. The longer H4⋯O1 contact in **MOP** (2.95 Å) is a result of the larger ring twist angle between the *para*-substituted phenyl ring and the amide linkage in **MOP**, 38.4° *versus* 31.4° in **TFMP**.

**Figure 5:**
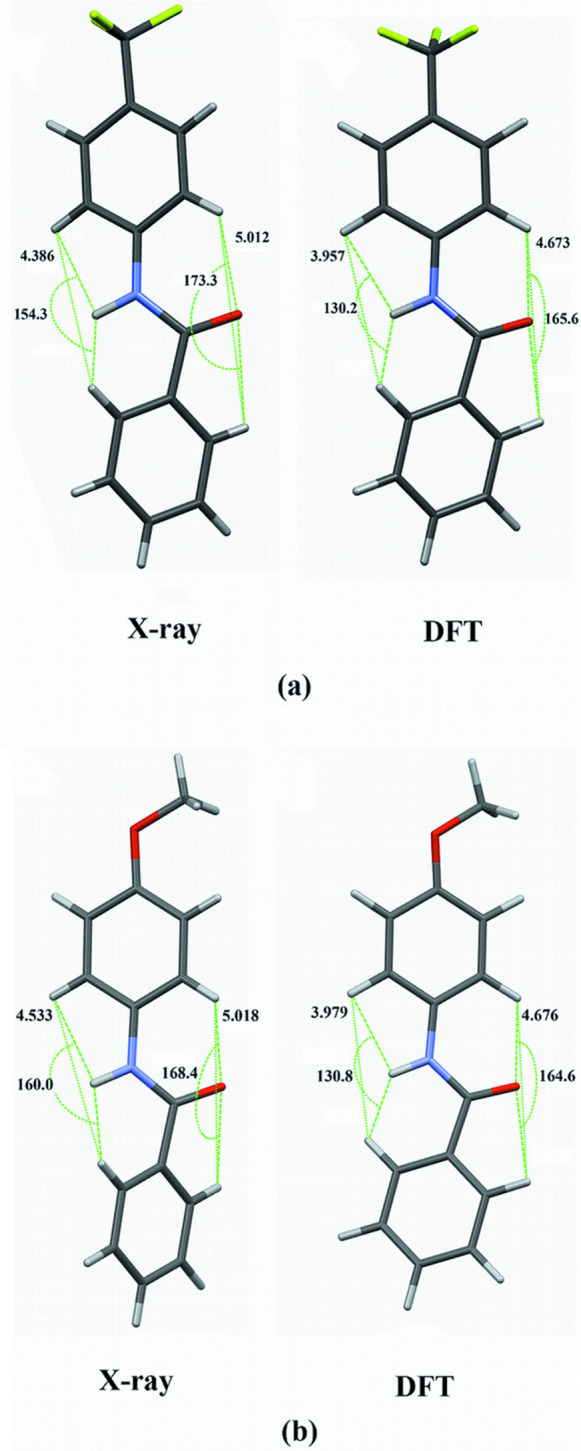
Comparison of hydrogen-bonding environments (in Å) from X-ray results and DFT calculations for (*a*) **TFMP** and (*b*) **MOP**.

Comparisons of hydrogen-bonding regions from the experimentally determined structure and DFT results are shown in Fig. 5 ▸ for **TFMP** and **MOP**. In both cases, the molecules in the crystal structures have a more open environment with larger angles around the donor and acceptor sites and larger donor and acceptor cavities. The increased planar tilt between *para*-substituted phenyl rings and amide planes is a contributor to the more open hydrogen-bonding environments in the experimentally determined structures.

**Figure 6:**
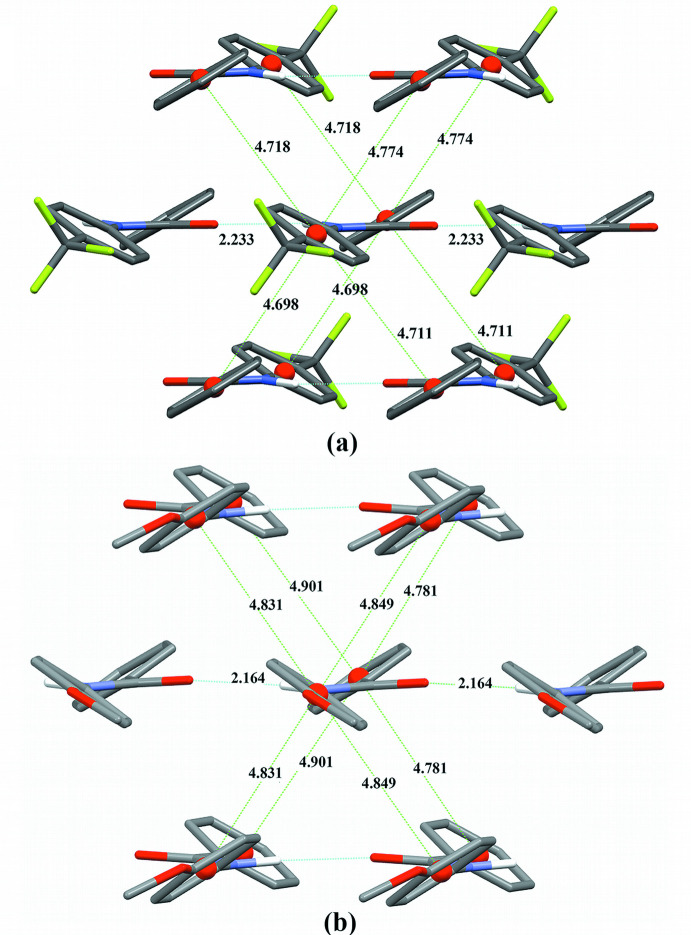
Hydrogen bonding and π-stacking (in Å) in (*a*) **TFMP** and (*b*) **MOP** (s.u.’s for centroid distances are approximately 0.005 Å). Riding H atoms are omitted for clarity.

The increased tilt angles between the amide and *para*-substituted phenyl planes also facilitate the π-stacking in both crystal structures (Table 3 ▸). Neighboring environments around aryl rings are shown in Fig. 6 ▸. Each aryl ring has close contacts with six other aryl rings. In **TFMP**, there are contacts between tri­fluoro­methyl­phenyl rings and phenyl rings. In **MOP**, phenyl rings have close contacts with phenyl rings while meth­oxy­phenyl rings have contacts with other meth­oxy­phenyl rings on neighboring mol­ecules. There are a total of six inter­actions surrounding each aryl ring, with four T-shaped inter­actions and two being a parallel displacement of rings. Neighboring mol­ecules that have parallel displaced rings are involved in the N—H⋯O hydrogen bonding. A qu­anti­tative discussion of the π stacking geometries based upon the approach of Banerjee *et al.* (2019 ▸) can be found in the supporting information.

**Figure 7:**
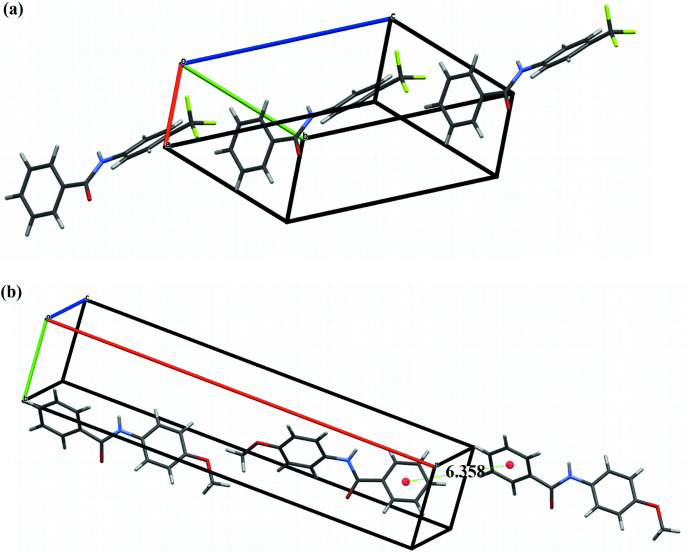
View of contacts (in Å) along the mol­ecular axes in (*a*) **TFMP** and (*b*) **MOP**.

Inter­molecular inter­actions in the remaining axial direction, *c* in **TFMP** and *a* in **MOP**, are shown in Fig. 7 ▸. In **TFMP**, the axial inter­actions are between a tri­fluoro­methyl group on one mol­ecule and a phenyl ring on its neighbor. In **MOP**, the closest inter­actions are of two types, meth­oxy­phen­yl–meth­oxy­phenyl inter­actions and phen­yl–phenyl inter­actions. The neighboring phenyl rings have a centroid distance of 6.4 Å and do not overlap.

## Hirshfeld surfaces and mol­ecular pair inter­action energies

To further examine the supra­molecular environments, Hirshfeld surfaces and mol­ecular pair inter­action energies were calculated for both crystal structures. All of these calculations were performed using the CE-B3LYP method *via* the *TONTO* program (Jayatilaka & Grimwood, 2003 ▸) as implemented in *CrystalExplorer17* (Spackman *et al.*, 2021 ▸). Inter­action energies use benchmarked models based upon B3LYP/6-31G(d,p) functionals, coupled with appropriate scale factors for electrostatic, polarization, dispersion and repulsion energies. The CE-B3LYP model is benchmarked against B3LYP-D2/6-31G(d,p) counterpoise-corrected energies and has been found to give very good agreement with CCSD(T)/CBS (Turner *et al.*, 2014 ▸).

**Figure 8:**
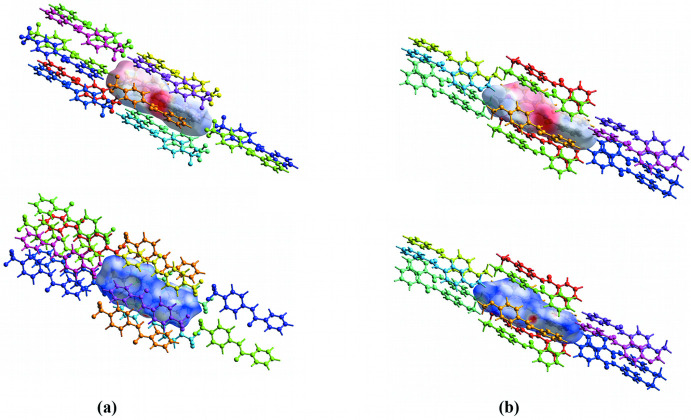
Inter­molecular inter­action energies and Hirshfeld surfaces with electrostatic potential (top) and *d*_norm_ (bottom) plots are shown for (*a*) **TFMP** and (*b*) **MOP**. Scales for electrostatic potential are red (−0.0788) to blue (0.1227) au for **TFMP** and red (−0.0875) to blue (0.1219) au for **MOP**. Scales for *d*_norm_ are red (−0.2905) to blue (0.9711) for **TFMP** and red (−0.3719) to blue (1.1524) for **MOP**. The color code for mol­ecular inter­actions is shown in Fig. 9 ▸.

**Figure 9:**
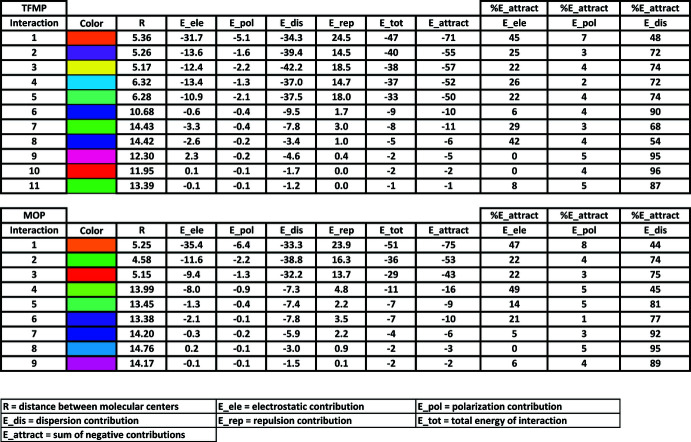
Key for the inter­molecular inter­action energies for **TFMP** and **MOP**. Energy units are kJ mol^−1^.

Hirshfeld surfaces and mol­ecular inter­action energies are shown in Fig. 8 ▸. The neighboring mol­ecules fall within 3.8 Å from the mol­ecule inside the Hirshfeld surface. The color coding keys and scaled energies are found in Fig. 9 ▸. Although the energy values are reported to 0.1 of a kJ mol^−1^, the authors of *CrystalExplorer17* recommend that the reliability is on the order of 1 kJ mol^−1^. As a result, the total inter­action energies (*E*_tot_) are rounded to a kJ mol^−1^. As expected, the major *E*_tot_ energies occur for the side-by-side inter­actions for **TFMP** (# 1–5) and **MOP** (# 1–3). The percent contributions to the *E*_attract_ from the electrostatic, polarization and dispersion components are reported. Dispersion is the major attractive inter­action in both crystal structures. For mol­ecules with hydrogen-bonded close contacts and for some inter­actions along the mol­ecular axes directions, the electrostatic energies are roughly equal to the dispersion energies. Videos showing 360° rotations of the static views in Fig. 8 ▸ can be found in the supporting information.

In Fig. 8 ▸, the electrostatic potentials, plotted on the Hirshfeld surfaces, show regions of negative charge (red) and positive charge (blue) for both compounds. For **TFMP**, in Fig. 8 ▸ *a*, the electrostatic inter­action of the hydrogen-bonding region is evident but so is the head-to-tail stacking of neighboring mol­ecules due to the attraction of negative tri­fluoro­methyl groups with neighboring positive phenyl hydrogens. For **MOP**, in Fig. 8 ▸ *b*, the electrostatic inter­action of the hydrogen bonding is apparent but the polar nature in the remaining segments of the mol­ecule is localized in the meth­oxy group, contributing to the preference for association of meth­oxy­phenyl rings in the crystal structure.

Fig. 8 ▸ also includes Hirshfeld surfaces with *d*_norm_ surface plots. Inter­molecular contacts less than a van der Waals contact are colored red, roughly equal contacts are white, and contacts longer than a van der Waals contact are blue. White or red contacts should indicate some degree of inter­molecular inter­action of inner and outer atoms at those positions on the Hirshfeld surfaces. Specific close contacts are shown in the supporting information.

Insight into the melting process for these crystals can be obtained from the energy analysis. Melting of these crystals would require overcoming the weak inter­molecular inter­actions along the direction of the mol­ecular axes. In the case of **TFMP**, the energy required would be on the order of 8–9 kJ mol^−1^ (inter­actions #6 and #7 in Fig. 9 ▸). In **MOP**, the energy required would only be around 7 kJ mol^−1^ (inter­actions #5 and #6). Although these energy values are inter­nal energies and not enthalpies, they are reasonable values for heats of fusion and correlate with the melting points of the two crystal structures, 478 K for **TFMP** and 425 K for **MOP** (Chang *et al.*, 2019 ▸). However, for **TFMP**, mol­ecules should separate equally at the melting point on either side of a mol­ecule. In **MOP**, mol­ecules will separate first at the phenyl ends of the mol­ecules while the meth­oxy­phenyl ends would be predicted to persist into the liquid phase until enough energy was applied to overcome the 11 kJ mol^−1^ inter­action energy (inter­action #4).

## Database survey

The Cambridge Structural Database was searched for possible crystal structures of these compounds. No entries were found for a crystal structure of *N*-[4-(tri­fluoro­meth­yl)phen­yl]benzamide. A room-temperature crystal structure was found for the *N*-(4-meth­oxy­phen­yl)benzamide compound (du Plessis *et al.*, 1983 ▸). The CIF file associated with this study, BUTDOJ, included only atom positions with no atomic displacement parameters. The *R* factor was listed as 0.106. In the published article, the authors noted that the overlapping reflections made it difficult to make an accurate background correction. This resulted, in the authors’ words, ‘in a somewhat poor set of intensity data for this compound’. For these reasons, we opted to use our redetermination of the crystal structure for the purpose of this publication.

## Synthesis and crystallization

Details of the synthesis of the title compounds can be found in Chang *et al.* (2019 ▸). Product crystals for both compounds were grown by slow diffusion of hexa­nes into a concentrated solution of the amide in ethyl acetate.

## Refinement

Crystal data, data collection and refinement details are summarized in Table 4 ▸. All hydrogen atoms were located in difference-Fourier maps. Final positions for most of the hydrogen atoms were calculated and included in a riding model relative to the bonded, non-hydrogen atoms by use of AFIX commands. Methyl hydrogen atoms were fixed at 0.98 Å from bonded carbon atoms, and phenyl hydrogen atoms were located 0.95 Å from bonded carbon atoms. Hydrogen displacement parameters were isotropic and set at 1.20 times the bonded phenyl carbons and 1.50 times the bonded methyl carbon in **MOP**. The amide hydrogens were treated differently because of their participation in the hydrogen bonding in these crystal structures. DFIX commands were set at 1.00 Å for these hydrogen atoms to allow for better comparison with the DFT-calculated N—H bond lengths. These hydrogen positions were then refined with independent isotropic displacement parameters. Isotropic extinction was refined in **MOP**. Although the ‘standard’ independent atom model was used for our analysis, alternative models were considered. Refinements with librationally corrected bond lengths and high-angle refinements were performed. These refinements had no significant effects on the structural results or the energy calculations.

## Supplementary Material

Crystal structure: contains datablock(s) global, 1, 2. DOI: 10.1107/S2056989022000950/wm5620sup1.cif

Structure factors: contains datablock(s) 1. DOI: 10.1107/S2056989022000950/wm56201sup2.hkl

Supporting information file. DOI: 10.1107/S2056989022000950/wm56201sup4.cml

Structure factors: contains datablock(s) 2. DOI: 10.1107/S2056989022000950/wm56202sup3.hkl

Supporting information file. DOI: 10.1107/S2056989022000950/wm56202sup5.cml

Pi stacking analysis. DOI: 10.1107/S2056989022000950/wm5620sup6.docx

Xray_DFT_bond length and angle comparisons. DOI: 10.1107/S2056989022000950/wm5620sup7.docx

close-ups of HS certain HS interactions. DOI: 10.1107/S2056989022000950/wm5620sup8.docx

Supporting information file. DOI: 10.1107/S2056989022000950/wm5620sup9.mp4

Supporting information file. DOI: 10.1107/S2056989022000950/wm5620sup10.mp4

Supporting information file. DOI: 10.1107/S2056989022000950/wm5620sup11.mp4

Supporting information file. DOI: 10.1107/S2056989022000950/wm5620sup12.mp4

CCDC references: 2144924, 2144923

Additional supporting information:  crystallographic information; 3D view; checkCIF report

## Figures and Tables

**Table 1:** Hydrogen-bond geometry (Å, °) for **TFMP** [Chem scheme1]

*D*—H⋯*A*	*D*—H	H⋯*A*	*D*⋯*A*	*D*—H⋯*A*
C6—H6⋯O1	0.95	2.44	2.938 (4)	112
C4—H4⋯O1^i^	0.95	2.57	3.240 (4)	128
N1—H1⋯O1^i^	0.99 (1)	2.23 (2)	3.138 (3)	151 (3)

Symmetry code: (i) 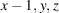 .

**Table 2:** Hydrogen-bond geometry (Å, °) for **MOP** [Chem scheme1]

*D*—H⋯*A*	*D*—H	H⋯*A*	*D*⋯*A*	*D*—H⋯*A*
C6—H6⋯O1	0.95	2.49	2.912 (2)	107
N1—H1⋯O1^i^	0.96 (1)	2.16 (1)	3.108 (2)	166 (2)

Symmetry code: (i) 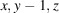 .

**Table 3:** π-stacking parameters for **TFMP** and **MOP** All distances are in Å with estimated uncertainties of 0.004. Angles are in ° with estimated uncertainties of 0.2.

Centroid	Normal	Offset	Twist angle
**TFMP** – surrounding both rings			
4.774	4.672	0.982	59.7
4.718	4.649	0.804	59.7
4.711	4.646	0.780	59.7
4.698	4.611	0.900	59.7
5.361	2.666	4.651	0.0
			
**MOP** – surrounding phenyl rings			
4.781	4.757	0.478	64.6
4.901	4.875	0.504	64.6
5.248	2.802	4.437	0.0
			
**MOP** – surrounding meth­oxy­phenyl rings			
4.849	4.658	1.348	68.1
4.831	4.64	1.345	68.1
5.248	2.938	4.349	0.0

**Table 4:** Experimental details

	**TFMP**	**MOP**
Crystal data		
Chemical formula	C_14_H_10_F_3_NO	C_14_H_13_NO_2_
*M*_r_	265.23	227.25
Crystal system, space group	Triclinic, *P* 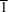	Monoclinic, *P*2_1_/*c*
Temperature (K)	173	173
*a*, *b*, *c* (Å)	5.3606 (11), 7.7831 (16), 14.415 (3)	26.7830 (15), 5.2477 (3), 8.1343 (5)
α, β, γ (°)	77.170 (7), 79.421 (7), 89.719 (7)	90, 97.594 (2), 90
*V* (Å^3^)	576.0 (2)	1133.24 (11)
*Z*	2	4
Radiation type	Mo *K*α	Mo *K*α
μ (mm^−1^)	0.13	0.09
Crystal size (mm)	0.24 × 0.08 × 0.06	0.70 × 0.26 × 0.08

Data collection		
Diffractometer	Bruker APEXII CCD	Bruker APEXII CCD
Absorption correction	Multi-scan (*SADABS*; Bruker, 2018 ▸)	Multi-scan (*SADABS*; Bruker, 2018 ▸)
*T*_min_, *T*_max_	0.700, 1.000	0.518, 1
No. of measured, independent and observed [*I* > 2σ(*I*)] reflections	11667, 2201, 1415	36318, 2333, 1678
*R*_int_	0.086	0.106
(sin θ/λ)_max_ (Å^−1^)	0.612	0.626

Refinement		
*R*[*F* ^2^ > 2σ(*F* ^2^)], *wR*(*F* ^2^), *S*	0.068, 0.189, 1.11	0.049, 0.126, 1.08
No. of reflections	2201	2333
No. of parameters	176	160
No. of restraints	1	1
H-atom treatment	H atoms treated by a mixture of independent and constrained refinement	H atoms treated by a mixture of independent and constrained refinement
Δρ_max_, Δρ_min_ (e Å^−3^)	0.26, −0.32	0.20, −0.19

Computer programs: *APEX3* and *SAINT* (Bruker, 2018 ▸), *SHELXT* (Sheldrick, 2015*a* ▸), *SHELXL* (Sheldrick, 2015*b* ▸), *WinGX* (Farrugia, 2012 ▸), *Mercury* (Macrae *et al.*, 2020 ▸), *CrystalExplorer17* (Spackman *et al.*, 2021 ▸), and *publCIF* (Westrip, 2010 ▸).

## supplementary crystallographic information

### N-[4-(Trifluoromethyl)phenyl]benzamide (1) Crystal data

**Table:**

C_14_H_10_F_3_NO	*F*(000) = 272
*M**_r_* = 265.23	*D*_x_ = 1.529 Mg m^−^^3^*D*_m_ = 1.46 (2) Mg m^−^^3^*D*_m_ measured by flotation in K2CO3(aq) solution
Triclinic, *P*1	Melting point = 477–478 K
*a* = 5.3606 (11) Å	Mo *K*α radiation, λ = 0.71073 Å
*b* = 7.7831 (16) Å	Cell parameters from 2253 reflections
*c* = 14.415 (3) Å	θ = 2.7–23.2°
α = 77.170 (7)°	µ = 0.13 mm^−^^1^
β = 79.421 (7)°	*T* = 173 K
γ = 89.719 (7)°	Regular parallelpiped, colourless
*V* = 576.0 (2) Å^3^	0.24 × 0.08 × 0.06 mm
*Z* = 2	

### N-[4-(Trifluoromethyl)phenyl]benzamide (1) Data collection

**Table:**

Bruker APEXII CCD diffractometer	2201 independent reflections
Radiation source: sealed X-ray tube	1415 reflections with *I* > 2σ(*I*)
Detector resolution: 8.53 pixels mm^-1^	*R*_int_ = 0.086
rotating crystal scans	θ_max_ = 25.8°, θ_min_ = 1.5°
Absorption correction: multi-scan (*SADABS*; Bruker, 2018)	*h* = −6→6
*T*_min_ = 0.700, *T*_max_ = 1.000	*k* = −9→9
11667 measured reflections	*l* = −17→17

### N-[4-(Trifluoromethyl)phenyl]benzamide (1) Refinement

**Table:**

Refinement on *F*^2^	Primary atom site location: structure-invariant direct methods
Least-squares matrix: full	Hydrogen site location: structure-invariant direct methods
*R*[*F*^2^ > 2σ(*F*^2^)] = 0.068	H atoms treated by a mixture of independent and constrained refinement
*wR*(*F*^2^) = 0.189	*w* = 1/[σ^2^(*F*_o_^2^) + (0.0726*P*)^2^ + 0.6085*P*] where *P* = (*F*_o_^2^ + 2*F*_c_^2^)/3
*S* = 1.11	(Δ/σ)_max_ < 0.001
2201 reflections	Δρ_max_ = 0.26 e Å^−^^3^
176 parameters	Δρ_min_ = −0.32 e Å^−^^3^
1 restraint	

### N-[4-(Trifluoromethyl)phenyl]benzamide (1) Special details

**Table:**

Geometry. All esds (except the esd in the dihedral angle between two l.s. planes) are estimated using the full covariance matrix. The cell esds are taken into account individually in the estimation of esds in distances, angles and torsion angles; correlations between esds in cell parameters are only used when they are defined by crystal symmetry. An approximate (isotropic) treatment of cell esds is used for estimating esds involving l.s. planes.

### N-[4-(Trifluoromethyl)phenyl]benzamide (1) Fractional atomic coordinates and isotropic or equivalent isotropic displacement parameters (Å^2^)

**Table:**

	*x*	*y*	*z*	*U*_iso_*/*U*_eq_	
C1	0.4290 (8)	0.2664 (5)	0.9270 (3)	0.0361 (10)	
C2	0.5101 (6)	0.2716 (5)	0.8223 (3)	0.0259 (8)	
C3	0.3572 (6)	0.3479 (5)	0.7572 (3)	0.0274 (9)	
H3	0.207249	0.403788	0.779424	0.033*	
C4	0.4208 (6)	0.3432 (5)	0.6611 (3)	0.0269 (8)	
H4	0.314467	0.395672	0.617458	0.032*	
C5	0.6407 (6)	0.2620 (4)	0.6270 (2)	0.0228 (8)	
C6	0.7958 (6)	0.1861 (5)	0.6926 (3)	0.0260 (8)	
H6	0.947277	0.131744	0.670270	0.031*	
C7	0.7301 (6)	0.1899 (5)	0.7885 (2)	0.0253 (8)	
H7	0.834978	0.136384	0.832438	0.030*	
C8	0.9209 (6)	0.2491 (5)	0.4709 (3)	0.0257 (8)	
C9	0.9138 (6)	0.2390 (5)	0.3692 (3)	0.0238 (8)	
C10	0.7131 (6)	0.1552 (5)	0.3439 (3)	0.0265 (8)	
H10	0.572074	0.103421	0.392070	0.032*	
C11	0.7225 (6)	0.1487 (5)	0.2481 (3)	0.0302 (9)	
H11	0.587155	0.091657	0.230932	0.036*	
C12	0.9254 (7)	0.2236 (5)	0.1773 (3)	0.0304 (9)	
H12	0.928932	0.218903	0.111750	0.036*	
C13	1.1249 (7)	0.3062 (5)	0.2021 (3)	0.0303 (9)	
H13	1.265418	0.357914	0.153574	0.036*	
C14	1.1180 (6)	0.3127 (5)	0.2979 (3)	0.0280 (9)	
H14	1.255099	0.368467	0.314711	0.034*	
F1	0.3071 (5)	0.4105 (3)	0.94336 (17)	0.0520 (7)	
F2	0.6234 (5)	0.2548 (4)	0.97444 (18)	0.0599 (8)	
F3	0.2713 (5)	0.1294 (4)	0.97311 (18)	0.0606 (8)	
N1	0.6886 (5)	0.2533 (4)	0.5288 (2)	0.0253 (7)	
O1	1.1224 (4)	0.2563 (4)	0.49980 (18)	0.0359 (7)	
H1	0.541 (5)	0.261 (6)	0.495 (3)	0.052 (13)*	

### N-[4-(Trifluoromethyl)phenyl]benzamide (1) Atomic displacement parameters (Å^2^)

**Table:**

	*U*^11^	*U*^22^	*U*^33^	*U*^12^	*U*^13^	*U*^23^
C1	0.039 (2)	0.033 (2)	0.037 (2)	0.0094 (19)	−0.0088 (18)	−0.0111 (18)
C2	0.0212 (17)	0.027 (2)	0.030 (2)	0.0040 (15)	−0.0059 (15)	−0.0067 (16)
C3	0.0187 (17)	0.031 (2)	0.033 (2)	0.0076 (15)	−0.0031 (15)	−0.0104 (17)
C4	0.0178 (17)	0.030 (2)	0.036 (2)	0.0046 (15)	−0.0092 (15)	−0.0095 (16)
C5	0.0179 (16)	0.022 (2)	0.029 (2)	0.0015 (14)	−0.0057 (14)	−0.0065 (15)
C6	0.0159 (17)	0.032 (2)	0.031 (2)	0.0082 (15)	−0.0052 (14)	−0.0077 (16)
C7	0.0204 (17)	0.027 (2)	0.029 (2)	0.0061 (15)	−0.0095 (15)	−0.0031 (15)
C8	0.0196 (18)	0.026 (2)	0.032 (2)	0.0036 (14)	−0.0044 (15)	−0.0080 (16)
C9	0.0191 (17)	0.024 (2)	0.0293 (19)	0.0114 (14)	−0.0054 (14)	−0.0070 (15)
C10	0.0158 (16)	0.029 (2)	0.035 (2)	0.0062 (14)	−0.0043 (14)	−0.0084 (16)
C11	0.0206 (18)	0.034 (2)	0.040 (2)	0.0090 (16)	−0.0123 (16)	−0.0127 (17)
C12	0.029 (2)	0.037 (2)	0.030 (2)	0.0127 (17)	−0.0098 (16)	−0.0119 (17)
C13	0.0233 (19)	0.033 (2)	0.032 (2)	0.0101 (16)	−0.0007 (15)	−0.0054 (17)
C14	0.0180 (17)	0.032 (2)	0.036 (2)	0.0071 (15)	−0.0073 (15)	−0.0097 (17)
F1	0.0738 (18)	0.0462 (16)	0.0401 (14)	0.0300 (13)	−0.0083 (12)	−0.0207 (12)
F2	0.0573 (16)	0.093 (2)	0.0404 (15)	0.0265 (15)	−0.0240 (13)	−0.0268 (14)
F3	0.0735 (19)	0.0528 (17)	0.0442 (16)	−0.0077 (14)	0.0159 (13)	−0.0089 (12)
N1	0.0147 (14)	0.0351 (19)	0.0279 (17)	0.0076 (13)	−0.0064 (12)	−0.0093 (13)
O1	0.0162 (12)	0.062 (2)	0.0331 (15)	0.0062 (12)	−0.0074 (11)	−0.0161 (13)

### N-[4-(Trifluoromethyl)phenyl]benzamide (1) Geometric parameters (Å, º)

**Table:**

C1—F3	1.335 (5)	C8—O1	1.233 (4)
C1—F2	1.340 (4)	C8—N1	1.370 (4)
C1—F1	1.340 (4)	C8—C9	1.493 (5)
C1—C2	1.484 (5)	C9—C14	1.384 (5)
C2—C3	1.391 (5)	C9—C10	1.405 (5)
C2—C7	1.397 (5)	C10—C11	1.385 (5)
C3—C4	1.373 (5)	C10—H10	0.9500
C3—H3	0.9500	C11—C12	1.378 (5)
C4—C5	1.396 (5)	C11—H11	0.9500
C4—H4	0.9500	C12—C13	1.391 (5)
C5—C6	1.403 (5)	C12—H12	0.9500
C5—N1	1.409 (4)	C13—C14	1.387 (5)
C6—C7	1.370 (5)	C13—H13	0.9500
C6—H6	0.9500	C14—H14	0.9500
C7—H7	0.9500	N1—H1	0.993 (10)
			
F3—C1—F2	105.9 (3)	O1—C8—N1	122.7 (3)
F3—C1—F1	106.1 (3)	O1—C8—C9	122.0 (3)
F2—C1—F1	105.7 (3)	N1—C8—C9	115.3 (3)
F3—C1—C2	112.7 (3)	C14—C9—C10	119.3 (3)
F2—C1—C2	113.1 (3)	C14—C9—C8	117.8 (3)
F1—C1—C2	112.7 (3)	C10—C9—C8	122.9 (3)
C3—C2—C7	119.0 (3)	C11—C10—C9	119.4 (3)
C3—C2—C1	120.0 (3)	C11—C10—H10	120.3
C7—C2—C1	120.9 (3)	C9—C10—H10	120.3
C4—C3—C2	120.7 (3)	C12—C11—C10	121.0 (3)
C4—C3—H3	119.7	C12—C11—H11	119.5
C2—C3—H3	119.7	C10—C11—H11	119.5
C3—C4—C5	120.5 (3)	C11—C12—C13	119.8 (3)
C3—C4—H4	119.8	C11—C12—H12	120.1
C5—C4—H4	119.8	C13—C12—H12	120.1
C4—C5—C6	118.9 (3)	C14—C13—C12	119.7 (3)
C4—C5—N1	117.8 (3)	C14—C13—H13	120.1
C6—C5—N1	123.3 (3)	C12—C13—H13	120.1
C7—C6—C5	120.4 (3)	C9—C14—C13	120.8 (3)
C7—C6—H6	119.8	C9—C14—H14	119.6
C5—C6—H6	119.8	C13—C14—H14	119.6
C6—C7—C2	120.6 (3)	C8—N1—C5	127.0 (3)
C6—C7—H7	119.7	C8—N1—H1	115 (3)
C2—C7—H7	119.7	C5—N1—H1	117 (3)

### N-[4-(Trifluoromethyl)phenyl]benzamide (1) Hydrogen-bond geometry (Å, º)

**Table:**

*D*—H···*A*	*D*—H	H···*A*	*D*···*A*	*D*—H···*A*
C6—H6···O1	0.95	2.44	2.938 (4)	112
C4—H4···O1^i^	0.95	2.57	3.240 (4)	128
N1—H1···O1^i^	0.99 (1)	2.23 (2)	3.138 (3)	151 (3)

Symmetry code: (i) *x*−1, *y*, *z*.

### N-(4-Methoxyphenyl)benzamide (2) Crystal data

**Table:**

C_14_H_13_NO_2_	*D*_x_ = 1.332 Mg m^−^^3^*D*_m_ = 1.29 (2) Mg m^−^^3^*D*_m_ measured by flotation in aqueous KI
*M**_r_* = 227.25	Melting point = 424–425 K
Monoclinic, *P*2_1_/*c*	Mo *K*α radiation, λ = 0.71073 Å
*a* = 26.7830 (15) Å	Cell parameters from 5514 reflections
*b* = 5.2477 (3) Å	θ = 3.1–26.0°
*c* = 8.1343 (5) Å	µ = 0.09 mm^−^^1^
β = 97.594 (2)°	*T* = 173 K
*V* = 1133.24 (11) Å^3^	Regular parallelpiped, colourless
*Z* = 4	0.70 × 0.26 × 0.08 mm
*F*(000) = 480	

### N-(4-Methoxyphenyl)benzamide (2) Data collection

**Table:**

Bruker APEXII CCD diffractometer	2333 independent reflections
Radiation source: sealed X-ray tube	1678 reflections with *I* > 2σ(*I*)
Detector resolution: 8.53 pixels mm^-1^	*R*_int_ = 0.106
rotating crystal scans	θ_max_ = 26.4°, θ_min_ = 1.5°
Absorption correction: multi-scan (*SADABS*; Bruker, 2018)	*h* = −33→33
*T*_min_ = 0.518, *T*_max_ = 1	*k* = −6→6
36318 measured reflections	*l* = −10→10

### N-(4-Methoxyphenyl)benzamide (2) Refinement

**Table:**

Refinement on *F*^2^	Hydrogen site location: structure-invariant direct methods
Least-squares matrix: full	H atoms treated by a mixture of independent and constrained refinement
*R*[*F*^2^ > 2σ(*F*^2^)] = 0.049	*w* = 1/[σ^2^(*F*_o_^2^) + (0.0247*P*)^2^ + 0.8083*P*] where *P* = (*F*_o_^2^ + 2*F*_c_^2^)/3
*wR*(*F*^2^) = 0.126	(Δ/σ)_max_ = 0.001
*S* = 1.08	Δρ_max_ = 0.20 e Å^−^^3^
2333 reflections	Δρ_min_ = −0.19 e Å^−^^3^
160 parameters	Extinction correction: SHELXL-2018/3 (Sheldrick, 2015b), Fc^*^=kFc[1+0.001xFc^2^λ^3^/sin(2θ)]^-1/4^
1 restraint	Extinction coefficient: 0.0099 (18)
Primary atom site location: structure-invariant direct methods	

### N-(4-Methoxyphenyl)benzamide (2) Special details

**Table:**

Geometry. All esds (except the esd in the dihedral angle between two l.s. planes) are estimated using the full covariance matrix. The cell esds are taken into account individually in the estimation of esds in distances, angles and torsion angles; correlations between esds in cell parameters are only used when they are defined by crystal symmetry. An approximate (isotropic) treatment of cell esds is used for estimating esds involving l.s. planes.
Refinement. isotropic extinction correction applied

### N-(4-Methoxyphenyl)benzamide (2) Fractional atomic coordinates and isotropic or equivalent isotropic displacement parameters (Å^2^)

**Table:**

	*x*	*y*	*z*	*U*_iso_*/*U*_eq_	
C1	0.53199 (8)	0.7401 (5)	0.1647 (3)	0.0544 (7)	
H1A	0.529249	0.745250	0.283557	0.082*	
H1B	0.498295	0.724788	0.101786	0.082*	
H1C	0.547952	0.897114	0.132379	0.082*	
C2	0.61069 (7)	0.5196 (4)	0.2084 (2)	0.0350 (5)	
C3	0.63993 (8)	0.3201 (4)	0.1635 (3)	0.0388 (5)	
H3	0.625795	0.197660	0.084749	0.047*	
C4	0.68972 (8)	0.2992 (4)	0.2334 (3)	0.0369 (5)	
H4	0.709605	0.161154	0.203434	0.044*	
C5	0.71075 (7)	0.4793 (4)	0.3471 (2)	0.0319 (4)	
C6	0.68127 (8)	0.6755 (4)	0.3937 (3)	0.0362 (5)	
H6	0.695284	0.796302	0.473747	0.043*	
C7	0.63122 (7)	0.6971 (4)	0.3240 (3)	0.0361 (5)	
H7	0.611137	0.833235	0.355679	0.043*	
C8	0.79514 (7)	0.6427 (4)	0.4517 (3)	0.0367 (5)	
C9	0.84768 (7)	0.5666 (4)	0.5223 (3)	0.0343 (5)	
C10	0.85869 (8)	0.3512 (4)	0.6189 (3)	0.0412 (5)	
H10	0.832343	0.240278	0.640884	0.049*	
C11	0.90810 (9)	0.2974 (5)	0.6833 (3)	0.0493 (6)	
H11	0.915465	0.151421	0.751318	0.059*	
C12	0.94644 (9)	0.4548 (5)	0.6491 (3)	0.0502 (6)	
H12	0.980294	0.416164	0.692292	0.060*	
C13	0.93588 (8)	0.6682 (5)	0.5525 (3)	0.0495 (6)	
H13	0.962451	0.776471	0.528736	0.059*	
C14	0.88664 (8)	0.7252 (4)	0.4899 (3)	0.0417 (5)	
H14	0.879440	0.873678	0.424217	0.050*	
N1	0.76211 (6)	0.4491 (3)	0.4173 (2)	0.0358 (4)	
O2	0.56174 (5)	0.5266 (3)	0.13011 (19)	0.0477 (4)	
O1	0.78412 (6)	0.8674 (3)	0.4262 (2)	0.0572 (5)	
H1	0.7748 (8)	0.277 (2)	0.424 (3)	0.041 (6)*	

### N-(4-Methoxyphenyl)benzamide (2) Atomic displacement parameters (Å^2^)

**Table:**

	*U*^11^	*U*^22^	*U*^33^	*U*^12^	*U*^13^	*U*^23^
C1	0.0349 (12)	0.0616 (16)	0.0642 (17)	0.0071 (11)	−0.0026 (11)	−0.0003 (13)
C2	0.0296 (10)	0.0395 (11)	0.0348 (11)	−0.0032 (9)	0.0001 (8)	0.0042 (9)
C3	0.0404 (12)	0.0366 (12)	0.0377 (12)	−0.0039 (9)	−0.0014 (9)	−0.0053 (9)
C4	0.0365 (11)	0.0321 (11)	0.0420 (12)	0.0014 (9)	0.0053 (9)	−0.0020 (9)
C5	0.0310 (10)	0.0309 (10)	0.0334 (11)	−0.0017 (8)	0.0024 (8)	0.0033 (8)
C6	0.0347 (11)	0.0341 (11)	0.0382 (12)	−0.0002 (9)	−0.0010 (9)	−0.0050 (9)
C7	0.0323 (11)	0.0356 (11)	0.0402 (12)	0.0021 (9)	0.0044 (9)	−0.0026 (9)
C8	0.0331 (11)	0.0313 (11)	0.0447 (13)	0.0016 (9)	0.0020 (9)	−0.0001 (9)
C9	0.0321 (11)	0.0316 (10)	0.0380 (11)	0.0013 (8)	0.0007 (9)	−0.0042 (9)
C10	0.0410 (12)	0.0359 (12)	0.0449 (13)	−0.0010 (9)	−0.0010 (10)	0.0002 (10)
C11	0.0505 (14)	0.0432 (13)	0.0504 (14)	0.0103 (11)	−0.0079 (11)	0.0015 (11)
C12	0.0349 (12)	0.0584 (15)	0.0539 (15)	0.0113 (11)	−0.0066 (10)	−0.0111 (12)
C13	0.0336 (12)	0.0557 (15)	0.0576 (15)	−0.0045 (11)	0.0009 (10)	−0.0062 (12)
C14	0.0365 (11)	0.0394 (12)	0.0479 (13)	−0.0023 (9)	0.0011 (10)	0.0016 (10)
N1	0.0297 (9)	0.0281 (9)	0.0479 (11)	0.0017 (7)	−0.0014 (7)	0.0007 (8)
O2	0.0301 (8)	0.0552 (10)	0.0548 (10)	0.0004 (7)	−0.0060 (7)	−0.0063 (8)
O1	0.0376 (9)	0.0302 (8)	0.0996 (14)	0.0016 (7)	−0.0063 (9)	0.0055 (9)

### N-(4-Methoxyphenyl)benzamide (2) Geometric parameters (Å, º)

**Table:**

C1—O2	1.424 (3)	C8—O1	1.227 (2)
C1—H1A	0.9800	C8—N1	1.352 (3)
C1—H1B	0.9800	C8—C9	1.502 (3)
C1—H1C	0.9800	C9—C10	1.386 (3)
C2—O2	1.380 (2)	C9—C14	1.387 (3)
C2—C7	1.385 (3)	C10—C11	1.387 (3)
C2—C3	1.385 (3)	C10—H10	0.9500
C3—C4	1.383 (3)	C11—C12	1.375 (3)
C3—H3	0.9500	C11—H11	0.9500
C4—C5	1.389 (3)	C12—C13	1.376 (3)
C4—H4	0.9500	C12—H12	0.9500
C5—C6	1.381 (3)	C13—C14	1.382 (3)
C5—N1	1.426 (2)	C13—H13	0.9500
C6—C7	1.389 (3)	C14—H14	0.9500
C6—H6	0.9500	N1—H1	0.965 (10)
C7—H7	0.9500		
			
O2—C1—H1A	109.5	O1—C8—C9	120.83 (18)
O2—C1—H1B	109.5	N1—C8—C9	115.72 (17)
H1A—C1—H1B	109.5	C10—C9—C14	119.21 (19)
O2—C1—H1C	109.5	C10—C9—C8	123.50 (19)
H1A—C1—H1C	109.5	C14—C9—C8	117.28 (19)
H1B—C1—H1C	109.5	C9—C10—C11	120.1 (2)
O2—C2—C7	124.29 (19)	C9—C10—H10	119.9
O2—C2—C3	115.73 (18)	C11—C10—H10	119.9
C7—C2—C3	119.98 (18)	C12—C11—C10	120.1 (2)
C4—C3—C2	119.97 (19)	C12—C11—H11	119.9
C4—C3—H3	120.0	C10—C11—H11	119.9
C2—C3—H3	120.0	C11—C12—C13	120.1 (2)
C3—C4—C5	120.28 (19)	C11—C12—H12	119.9
C3—C4—H4	119.9	C13—C12—H12	119.9
C5—C4—H4	119.9	C12—C13—C14	120.0 (2)
C6—C5—C4	119.58 (18)	C12—C13—H13	120.0
C6—C5—N1	121.95 (18)	C14—C13—H13	120.0
C4—C5—N1	118.42 (18)	C13—C14—C9	120.4 (2)
C5—C6—C7	120.34 (19)	C13—C14—H14	119.8
C5—C6—H6	119.8	C9—C14—H14	119.8
C7—C6—H6	119.8	C8—N1—C5	124.71 (17)
C2—C7—C6	119.81 (19)	C8—N1—H1	118.4 (13)
C2—C7—H7	120.1	C5—N1—H1	116.2 (13)
C6—C7—H7	120.1	C2—O2—C1	116.87 (17)
O1—C8—N1	123.44 (19)		

### N-(4-Methoxyphenyl)benzamide (2) Hydrogen-bond geometry (Å, º)

**Table:**

*D*—H···*A*	*D*—H	H···*A*	*D*···*A*	*D*—H···*A*
C6—H6···O1	0.95	2.49	2.912 (2)	107
N1—H1···O1^i^	0.96 (1)	2.16 (1)	3.108 (2)	166 (2)

Symmetry code: (i) *x*, *y*−1, *z*.
