# Supplementary material for: Crystal structures of N-[4-(tri­fluoro­meth­yl)phen­yl]benzamide and N-(4-meth­oxy­phen­yl)benz­amide at 173 K: a study of the energetics of conformational changes due to crystal packing
Source: Acta Crystallogr E Crystallogr Commun. 2022 Feb 8;78(Pt 3):297–305. doi: 10.1107/S2056989022000950 (PMC8900516; doi:10.1107/S2056989022000950)
Supplement: Supplementary file 6 [file e-78-00297-sup6.docx]

Detailed look at the geometry of pi stacking interactions:

One of the pi stacking interactions for **TFMP** is shown. The major parameters in the interaction are the centroid distance (4.774 Å) the normal between the centroid of the trifluoromethyl ring and the plane of the bottom phenyl ring (4.672 Å) and the tilt angle of the two rings (59.7 degrees). Completing the right triangle between the two rings allows for a calculation of the offset of the two rings as 0.982 A. Tabulations of these parameters for both structures are given in the accompanying table. An extensive study of pi stacking geometries for structures in the literature has been published (Banerjee *et al.* (2019)) . According to this study, the geometries of the pi stacking interactions shown in Table 3 for both **TFMP** and **MOP** are commonly found in other benzenoid structures.

Banerjee, A., Saha, A. & Saha, B. K. (2019). *Cryst. Growth Des.* **19**, 2245-2252.


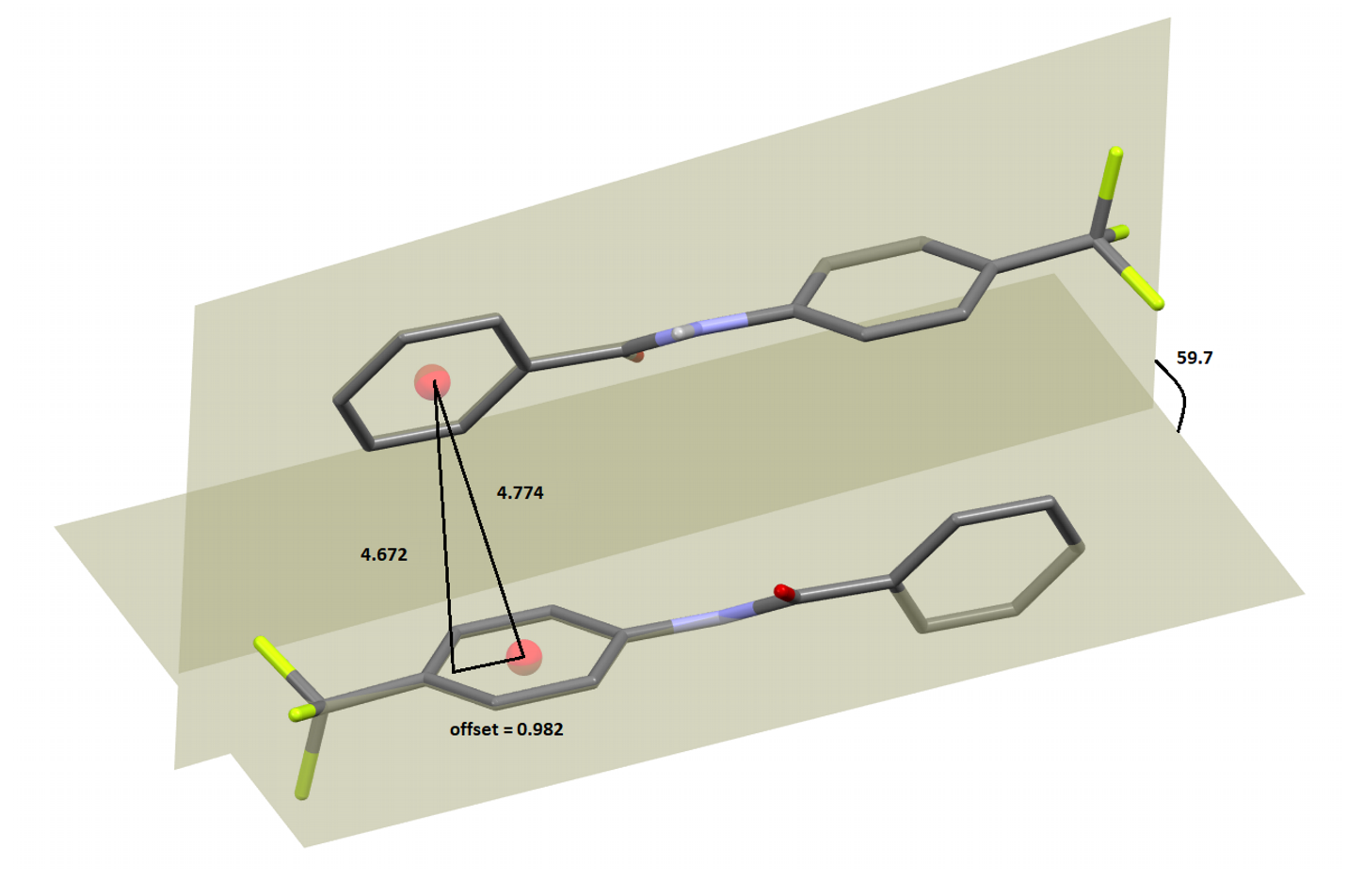


"Geometry of pi stacking showing centroid distance, 4.774, and plane normal

distance, 4.672, in the calculation of the offset distance, 0.982. All

distances are in Å.

| [**TFMP**- surrounding both rings](global%20_geom_extra_tableA_col_1) |  |  |  |
| --- | --- | --- | --- |
| [Centroid](global%20_geom_extra_tableA_col_1) | [Normal](global%20_geom_extra_tableA_col_1) | [Offset](global%20_geom_extra_tableA_col_1) | [Twist angle](global%20_geom_extra_tableA_col_1) |
| [4.774](global%20_geom_extra_tableA_col_1) | [4.672](global%20_geom_extra_tableA_col_1) | [0.982](global%20_geom_extra_tableA_col_1) | [59.7](global%20_geom_extra_tableA_col_1) |
| [4.718](global%20_geom_extra_tableA_col_1) | [4.649](global%20_geom_extra_tableA_col_1) | [0.804](global%20_geom_extra_tableA_col_1) | [59.7](global%20_geom_extra_tableA_col_1) |
| [4.711](global%20_geom_extra_tableA_col_1) | [4.646](global%20_geom_extra_tableA_col_1) | [0.780](global%20_geom_extra_tableA_col_1) | [59.7](global%20_geom_extra_tableA_col_1) |
| [4.698](global%20_geom_extra_tableA_col_1) | [4.611](global%20_geom_extra_tableA_col_1) | [0.900](global%20_geom_extra_tableA_col_1) | [59.7](global%20_geom_extra_tableA_col_1) |
| [5.361](global%20_geom_extra_tableA_col_1) | [2.666](global%20_geom_extra_tableA_col_1) | [4.651](global%20_geom_extra_tableA_col_1) | [0.0](global%20_geom_extra_tableA_col_1) |
| [**MOP** - surrounding phenyl rings](global%20_geom_extra_tableA_col_1) |  |  |  |
| [Centroid](global%20_geom_extra_tableA_col_1) | [Normal](global%20_geom_extra_tableA_col_1) | [Offset](global%20_geom_extra_tableA_col_1) | [Angle](global%20_geom_extra_tableA_col_1) |
| [4.781](global%20_geom_extra_tableA_col_1) | [4.757](global%20_geom_extra_tableA_col_1) | [0.478](global%20_geom_extra_tableA_col_1) | [64.6](global%20_geom_extra_tableA_col_1) |
| [4.901](global%20_geom_extra_tableA_col_1) | [4.875](global%20_geom_extra_tableA_col_1) | [0.504](global%20_geom_extra_tableA_col_1) | [64.6](global%20_geom_extra_tableA_col_1) |
| [5.248](global%20_geom_extra_tableA_col_1) | [2.802](global%20_geom_extra_tableA_col_1) | [4.437](global%20_geom_extra_tableA_col_1) | [0.0](global%20_geom_extra_tableA_col_1) |
| [**MOP** - surrounding methoxyphenyl rings](global%20_geom_extra_tableA_col_1) |  |  |  |
| [Centroid](global%20_geom_extra_tableA_col_1) | [Normal](global%20_geom_extra_tableA_col_1) | [Offset](global%20_geom_extra_tableA_col_1) | [Angle](global%20_geom_extra_tableA_col_1) |
| [4.849](global%20_geom_extra_tableA_col_1) | [4.658](global%20_geom_extra_tableA_col_1) | [1.348](global%20_geom_extra_tableA_col_1) | [68.1](global%20_geom_extra_tableA_col_1) |
| [4.831](global%20_geom_extra_tableA_col_1) | [4.64](global%20_geom_extra_tableA_col_1) | [1.345](global%20_geom_extra_tableA_col_1) | [68.1](global%20_geom_extra_tableA_col_1) |
| [5.248](global%20_geom_extra_tableA_col_1) | [2.938](global%20_geom_extra_tableA_col_1) | [4.349](global%20_geom_extra_tableA_col_1) | [0.0](global%20_geom_extra_tableA_col_1) |
