# Supplementary figures and images for: Crystal structures of N-[4-(tri­fluoro­meth­yl)phen­yl]benzamide and N-(4-meth­oxy­phen­yl)benz­amide at 173 K: a study of the energetics of conformational changes due to crystal packing
Source: Acta Crystallogr E Crystallogr Commun. 2022 Feb 8;78(Pt 3):297–305. doi: 10.1107/S2056989022000950 (PMC8900516; doi:10.1107/S2056989022000950)

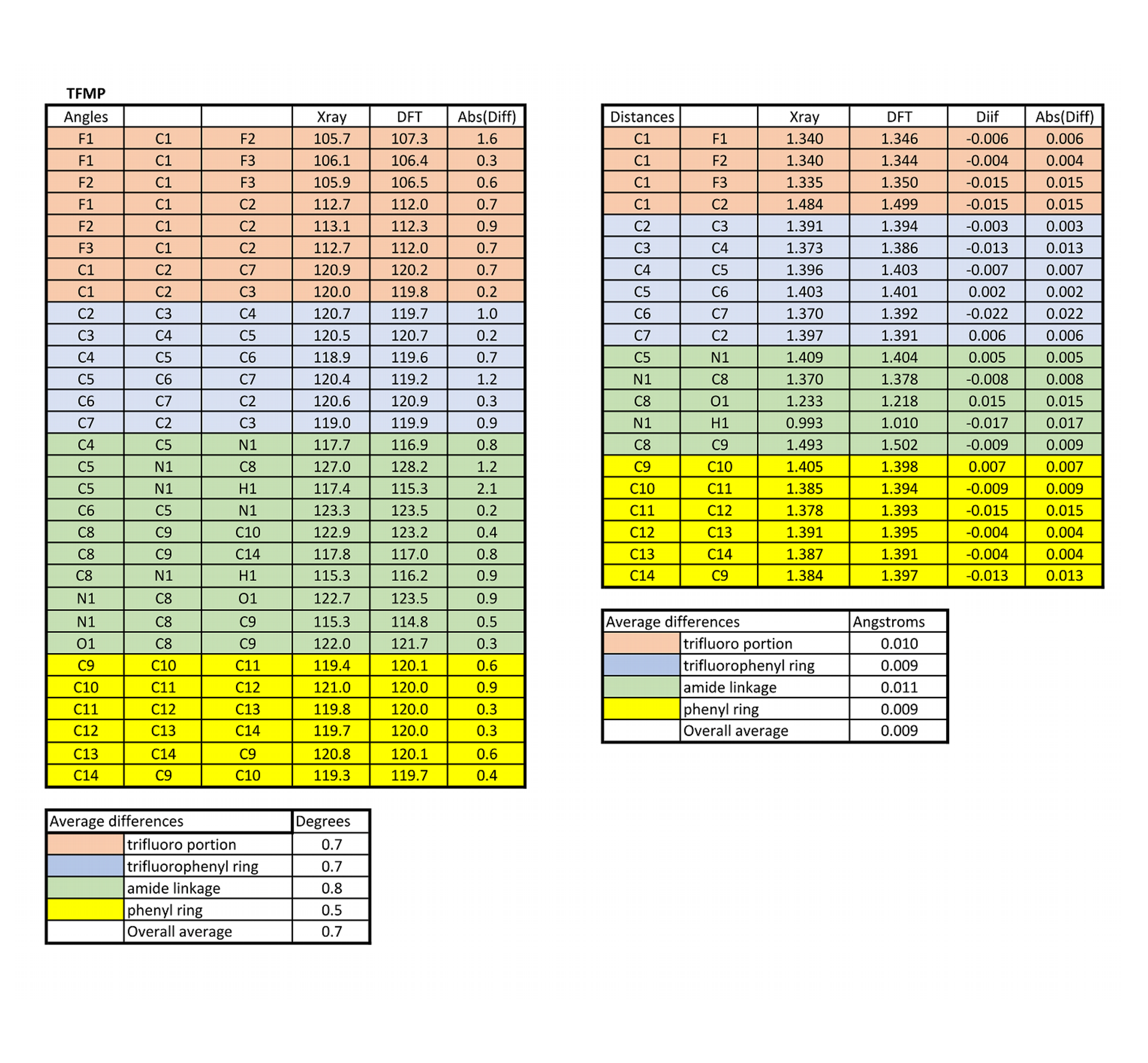


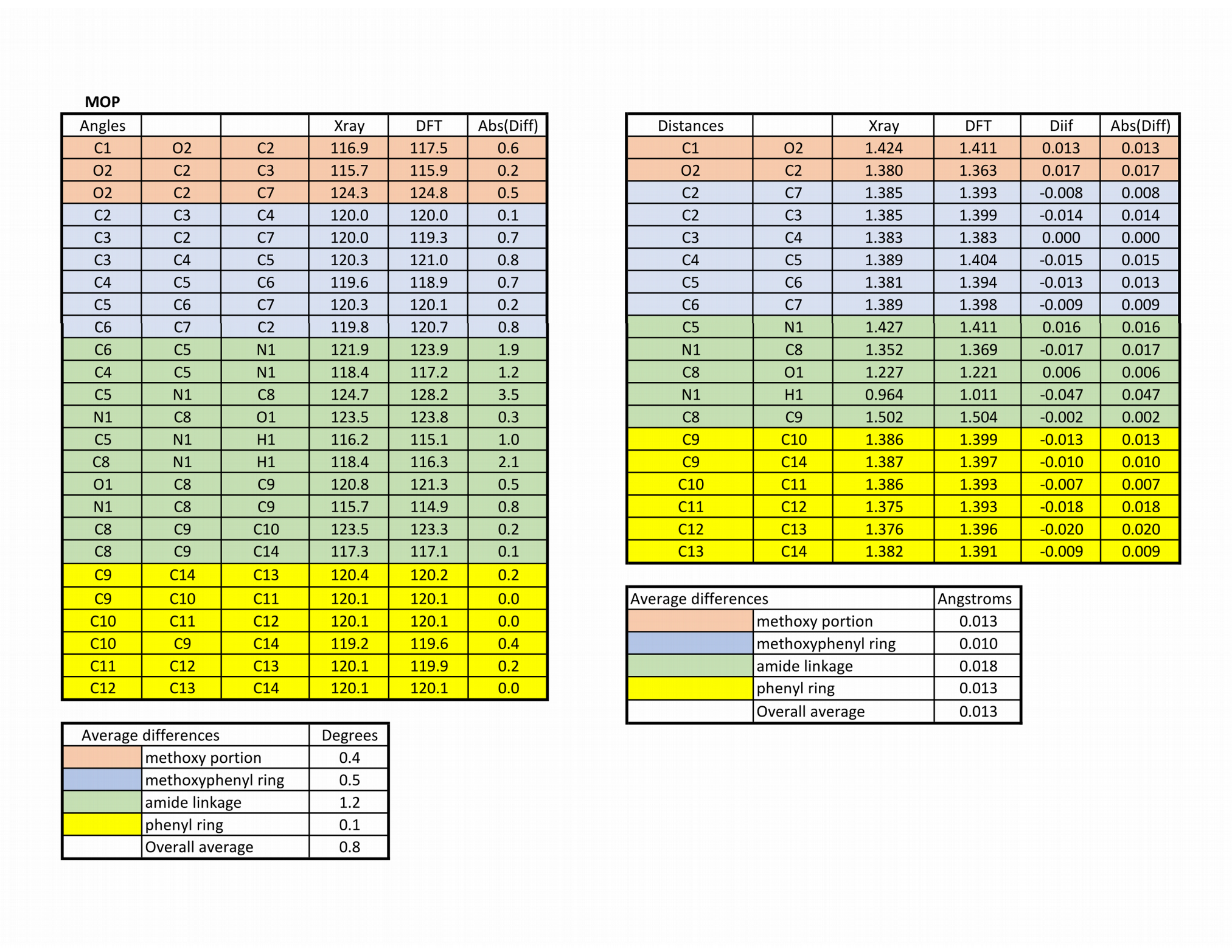

Supplement: Supplementary file 7 [file e-78-00297-sup7.docx]
