# Supplementary material for: Crystal structures of N-[4-(tri­fluoro­meth­yl)phen­yl]benzamide and N-(4-meth­oxy­phen­yl)benz­amide at 173 K: a study of the energetics of conformational changes due to crystal packing
Source: Acta Crystallogr E Crystallogr Commun. 2022 Feb 8;78(Pt 3):297–305. doi: 10.1107/S2056989022000950 (PMC8900516; doi:10.1107/S2056989022000950)
Supplement: Supplementary file 8 [file e-78-00297-sup8.docx]

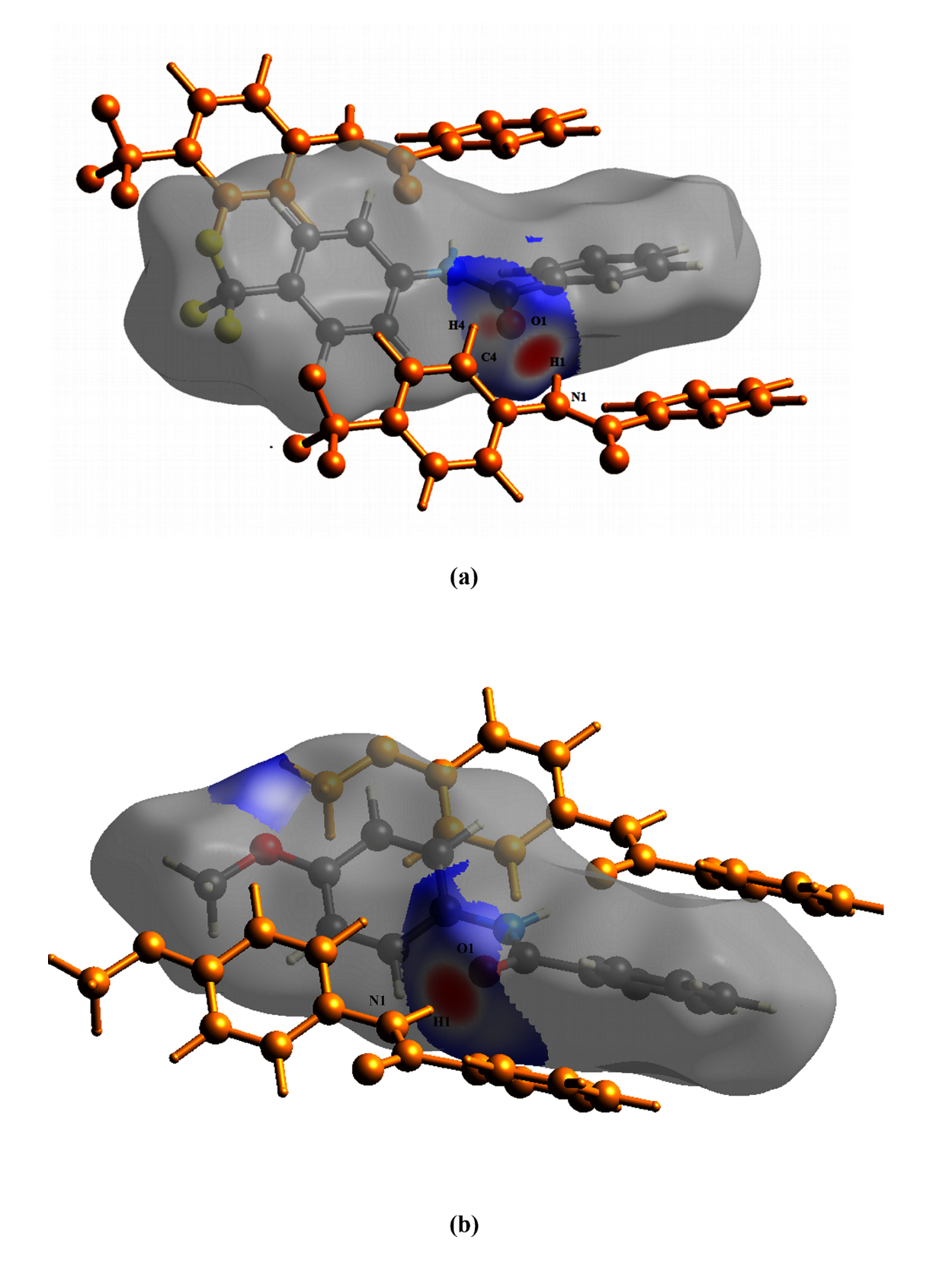


Isolated O-H contacts in (a) **TFMP** and (b) **MOP**. Hydrogen bonding is shown by isolating d_norm_ values between inner oxygen atoms and external hydrogens.


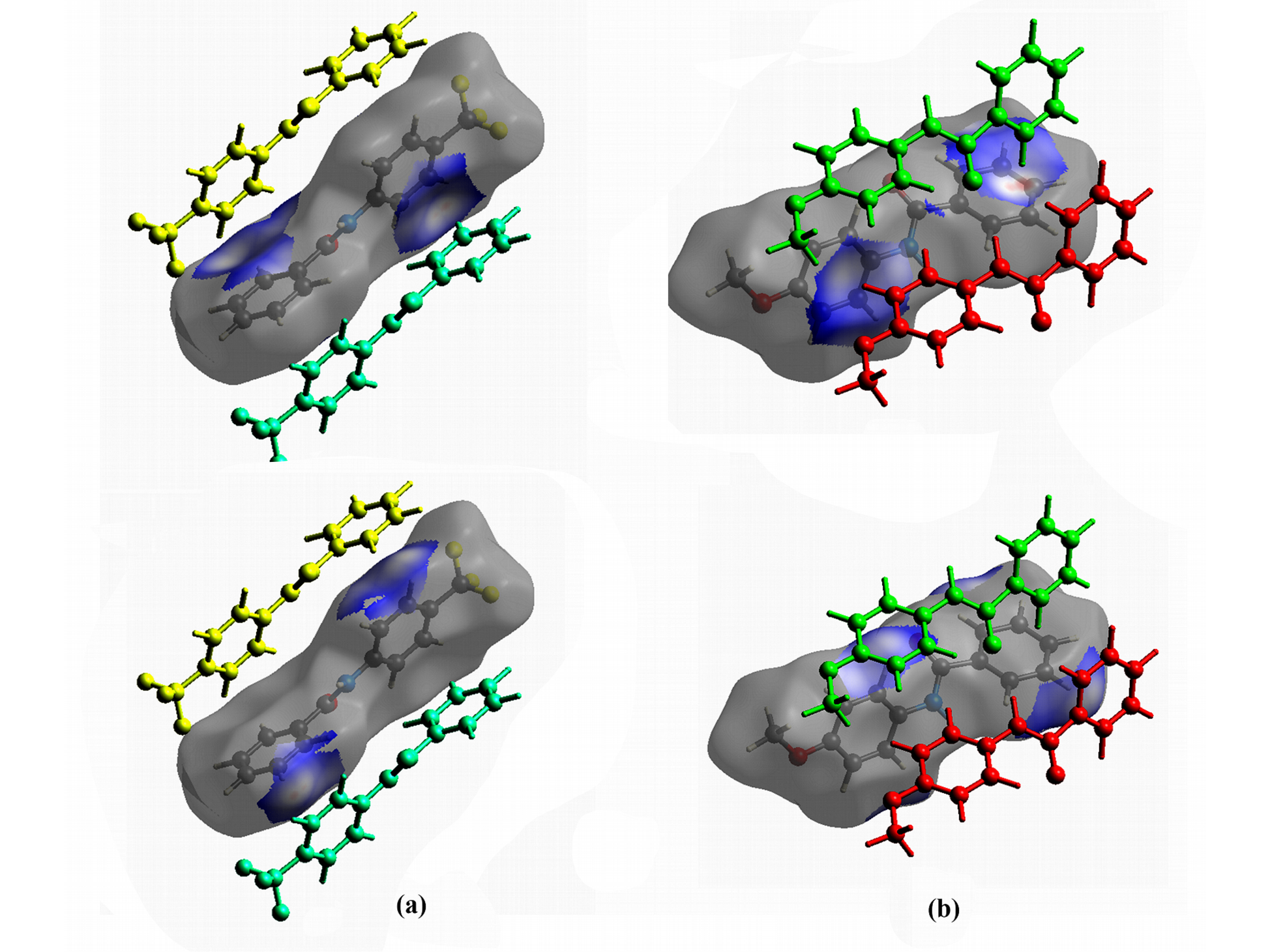


C-H to π cloud interactions in (a) **TFMP** and (b) **MOP**


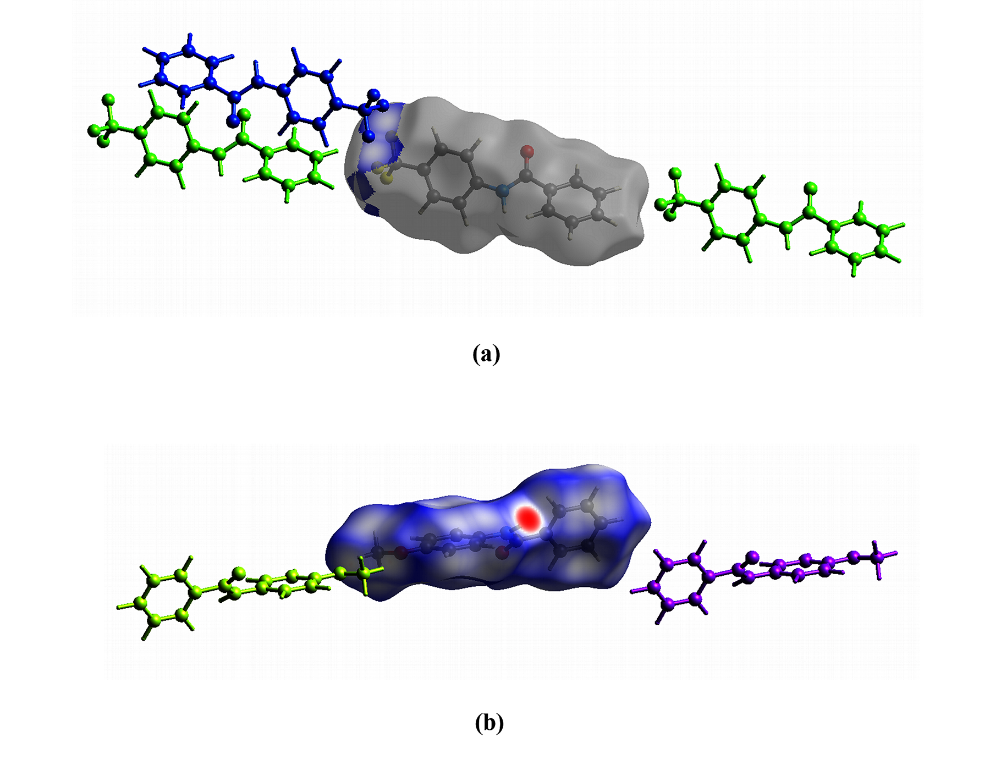


The close contacts of fluorines are shown as primary contributors to the dispersion energy that dominates interaction energy #6 in (a) **TFMP**. This dispersion energy is the largest interaction in the direction of the molecular axes. This close contact may be responsible for the ordering of fluorines in the crystal structure instead of the rather common six-fold disorder found in X-ray structures involving trifluoromethyl groups. The methoxy group alignment in the foreground and the phenyl H-H contacts in the background are shown for (b) **MOP**.
